# Survival of neonates and predictors of their mortality in Tigray region, Northern Ethiopia: prospective cohort study

**DOI:** 10.1186/s12884-016-0994-9

**Published:** 2016-08-02

**Authors:** Hayelom Gebrekirstos Mengesha, Alem Desta Wuneh, Wondwossen Terefe Lerebo, Tesfay Hailu Tekle

**Affiliations:** 1 Adigrat University, College of Medicine and Health science, Adigrat, Ethiopia; 2 Mekelle University, College of Health Science, Mekelle, Ethiopia

**Keywords:** Ethiopia, Neonatal mortality, Survival analysis, Tigray region

## Abstract

**Background:**

Neonatal mortality accounts for an estimated 2.8 million deaths worldwide, which constitutes 44 % of under-5-mortality and 60 % of infant mortality. Neonatal mortality predictors vary by country with the availability and quality of health care. Therefore, aim of this study was to estimate survival time and identify predictors of neonatal mortality in Tigray region, northern Ethiopia.

**Method:**

A prospective cohort study design was carried out among a cohort of neonates delivered in seven hospitals of Tigray from April to July, 2014 and followed up for a total of 28 days. Data were collected by interviewing mothers using structured questionnaires and assessments of the neonate and mothers by midwives. Kaplan-Meier, Log rank test and Cox-proportional hazard regressions were used. STATA V-11 program was used for data entry, cleaning and analysis.

**Results:**

From 1152 neonates, 68 died (neonatal mortality rate 62.5/1000 live births), 73.52 % of the neonates died within 7 days, 60 were lost to follow-up and the percentage of survival at 28 days was 93.96 % (95 % CI: 92.4, 95.2 %). Predictors of neonatal mortality were: normal birth weight (AHR: 0.45, 95 % CI: 0.24, 0.84), not initiating exclusive breastfeeding (AHR: 7.5, 95 % CI: 3.77, 15.05), neonatal complications (AHR: 0.14, 95 % CI 0.07, 0.29), maternal complications (AHR: 0.37, 95 % CI: 0.22, 0.63) and proximity (AHR: 2.5, 95 % CI: 1.29, 4.91).

**Conclusion:**

Neonatal mortality is unacceptably very high. Managing complications and low birth weight, initiating exclusive breast feeding, improving quality of services and ensuring a continuum of care are recommended to increase survival of neonates.

## Background

Neonatal mortality (NM) accounts for an estimated 2.8 million deaths worldwide [1], which constitute 44 % of child mortality and over 60 % of infant mortality [1, 2]. Most of these (99 %) arise in low-income and middle-income countries [3]. Hence, special endeavors should be undertaken to reduce the proportion of NM in under-five mortality (U5M). Sub-Saharan Africa carries the highest NM in the world and made the lowest progress in reducing NM [1, 2]. In Ethiopia, the proportion of NM in U5M became 42 % in 2011 [4]. A recent report in 2014 indicates that Ethiopia has achieved Millennium Development Goal-4 in 2012 [1] and an increasing number of the remaining child mortalities are attributed to NM intrinsically linked to maternal health and nutrition [5]. Ethiopia’s achievements have been attributed to the country community-based health promotion and disease prevention strategies [6]. In addition, for years, Ethiopia has been implementing integrated services of Emergency Obstetric and Newborn Care to improve neonatal and maternal care [7, 8] and to reduce neonatal deaths. Ethiopia neonatal mortality rate (NMR) in 2011 was 37/1000 [4, 9] and this rate varies by region from 21–62/1000 live births. The Tigray region (in which the study was conducted) NMR was 44/1000 [4].

High maternal and neonatal mortality and still birth often occur because of inadequate care during pregnancy and childbirth. Hence, these deaths are considered as sensitive indicators of the quality of the healthcare system in the area [10]. One-third to half of NM occurred within 24-h and 75 % occurred in the first week of life [1, 3, 11, 12] because of complications during pregnancy and childbirth [1–3]. Many studies have revealed NM is influenced by multiple factors [3, 13–16]. Previous reports in Ethiopia showed the causes of NM as sepsis, asphyxia, birth injury, tetanus, preterm birth, congenital malformations and unknown causes [17]. However, these reports are either hospital based secondary records [18] or retrospective household surveys [4].

Furthermore, causes of NM vary by country and region with the availability and quality of health care, therefore, understanding NM in relation to these factors is crucial [3, 19, 20]. This is because there are highly feasible and cost-effective interventions that could avert NM up to 72 %, and this can only be achieved if countries adopt locally relevant and focused interventions that are guided by evidence [1, 3]. Considering the paucity of reliable and documented evidence on NM in the study region, Tigray, we aimed at clearly identifying predictors and estimating survival time of neonates delivered in hospitals of Tigray region, which have access to skilled birth and obstetric care. This included all proximate and distal determinants of NM and following up prospectively from birth up to 28 days.

## Methods

### Study design, setting and population

A prospective cohort study was conducted among a cohort of neonates delivered between April 2014 and July 2014 in randomly selected seven hospitals of Tigray region, which is one of the nine administrative Federal regions of Ethiopia. Tigray region has an estimated total population of 4,565,000, (2,314,000 women and 2,251,000 men). The Ethiopian health care has a decentralized four-tier system of primary, district, zonal and specialized hospitals. The primary care includes rural health posts, nested into health centers serving 25,000 populations. District hospitals form the tiers expected to serve 250,000 catchment populations. Zonal and specialized hospital serves 500,000 and 5,000,000 populations respectively. In Tigray region, one of the 11 administrative states in Ethiopia, has a total of 15 public hospitals; 6 zonal, 1 specialized hospitals, and the remaining 8 in the district and primary health care category. All hospitals provide maternal and newborn services including delivery services free of charge. Seven hospitals were randomly selected from the 15 hospitals of the region by lottery method [21]. All mothers who gave a live birth in the study hospitals came from the entire region were included in this study and followed up for a total of 28 days.

### Inclusion and exclusion criteria

Mothers with their Neonates, who gave live birth in the study hospitals or admitted within six hours, from April -July, 2014 were included in the study. Mothers who were unable to speak and mothers with psychiatric illnesses were excluded from the study.

### Sample size

The sample size was 1162 neonates, which were determined by using STATA statistical package, Version 11.0. Based on the assumptions of 2.19 hazard-ratio [22] associated preceding birth of the mother, there was a variability of 0.5, probability of death 0.044 [4], 5 % marginal error and 95 % confidence interval of certainty to have a power of 80 %. It was assumed that no subjects were anticipated to withdraw from the follow-up.

### Data collection, participants, recruitment and follow-up

Data were collected prospectively using pre-tested structured questionnaires and using a checklist for data that were collected from assessments of the neonate and mother. The questionnaire was derived from related literatures and WHO standard verbal autopsy questionnaires [23]. The questionnaire was initially developed in English and translated into local language, *Tigrigna*. Two midwives, with a qualification of university degree and can speak the local language from each hospital were recruited for data collection. The data collectors had been collecting information by interviewing all mothers who delivered a live birth at the selected hospitals within the first 6-h after delivery. Besides, the clinical information was extracted by assessment of the neonate and mother. Mothers and live births were followed for 28 days, using three alternatives. The data collector visited the neonate daily, while he/she was in the hospital. After the mother was discharged; the data collector met the mother every 7-days either using a phone call or by creating liaison with a health extension worker and inquired about the neonatal condition and survival. Mothers who were not at home during the scheduled visit were revisited. When death occurred, the date and cause of death was recorded and probable cause of death was assigned after agreement between midwives and physicians based on national guidelines [24] and the International classification of disease [25]. The data that were collected prospectively consisted of Time to death of neonates (Dependent variable), and the socio-demographic, economical, neonatal, and maternal and health service-related characteristics (independent variables).

### Definition and measurement of variables

The inclusion of variables was partly guided by the Mosley and Chen conceptual framework [26] previously used in similar studies with some modifications. The factors influencing NM were broadly categorized as follows:

### Distal (socioeconomic and demographic factors)

The variables assessed in this category were: monthly reported income categorized into, low (<500 ETB), medium (500–1500 ETB) and rich (>2000ETB), and residence classified into ‘rrban’/‘rural’. Age at first marriage was measured in continuous scale yet for the sake of analysis later it was categorized into below age of 18 (teenagers), ‘18 years and above’. Similarly, educational status was first categorized in to ‘unable to read and write’,’able to read and write but no formal education’, ‘1–4 grade’, ‘5–8 grade’, ‘9–12 grade’, ‘college and above’ but later during analysis considering the few number of observations in the ‘able to read & write’ category, we merged it into (unable to read & write with able to read & write), and finally categorized into : ‘(unable to write & read or no formal education)’, ‘primary education (1–8 grade)’, ‘secondary (9–12 grade)’ and ‘tertiary (college and above grades)’.

Likewise, marital status was first categorized into married (living together), single, widowed, divorced and separated but later during analysis due to few observations, we merged the categories other than the ‘married’ category in to ‘others’. Occupation was classified based on the mothers report into ‘house wife/farmer’, ‘own business’ ‘government employee’ and ‘student’.

### Proximal factors (maternal, neonatal and health service related)

This category includes: Gestational age, which was measured by using last menstrual period and/or ultrasound. Gestational age was categorized into preterm (<259 days), term (259–293 days) and post term birth (> = 294 days) but during analysis due to the few observations of post term births coded with term birth. Birth weight was defined according to the WHO classification as low birth weight (<2500 gm); normal birth weight (> = 2500 and <4000 gm); and macrosomia (> = 4000 gm) [27] but later during analysis, it was coded into low birth weight and normal birth weight (> = 2500 gm) and it was measured using a standard beam balance within six hours of delivery.

Body mass index (BMI), measured by a standard height, weight measuring instrument found in the study hospitals and was categorized into underweight (BMI less than 18.5), normal (BMI 18.5–24.9), Overweight (BMI ≥25.0). Weight for gestational age was categorized according to WHO definitions into: ‘Large for gestational age’ was defined as a birth weight greater than the 90^th^ percentile for age, ‘Small for gestational age’ birth weights below the 10th percentile. ‘Appropriate for gestational age’ defined as birth weight between the 90–10th percentile [28]. The cut off points of weight for gestational age was derived from Alexander et al. described elsewhere [29] and adapted to our study with some modifications.

In our study, fertility (no of children) defined as the actual production of live offspring excluding stillbirths, fetal deaths and abortions. It was classified into primiparae (only the current birth), 2–4 and multiparty (> = 5). But parity was defined as the number of full term children previously borne by a woman, excluding miscarriages or abortions in early pregnancy, but in contrast to fertility it includes stillbirths. It was categorized into ‘none’ ‘one’ ‘two’ and ‘> = three’.

We defined gravidity as the number of pregnancies (completed or incomplete) experienced by a woman in contrast to fertility and gravidity it includes all pregnancies. We categorized it into ‘no pregnancy’, ‘one pregnancy’, ‘two pregnancy’ and ‘> = three pregnancy’.

Antenatal follow up was assessed by categorizing in to ‘Yes’, if the mother ever had at least one antenatal follow up, ‘No’ if the mother never had antenatal follow up.

Diagnosed medical disease was assessed using, ‘Yes’ if the mother had a medically known/diagnosed disease such as HIV/AIDS, Hypertension, Diabetes mellitus, epilepsy and ‘others’. ‘No’, if the mother did not have medically diagnosed disease.

History abortion defined as mother history of abortion either induced, spontaneous or self initiated abortion before the current birth. It was categorized into ‘Yes’/‘No’.

Induced abortion was defined as an abortion induced medically by a health professional, but self induced abortion was defined as abortion initiated by the women without medical and legal indication. Spontaneous abortion was defined as an abortion, which is neither medically induced nor self initiated, but the fetus spontaneously aborted itself. Similarly, history of still birth was categorized into ‘Yes’ and ‘No’.

Place of birth (inborn or out born) was defined ‘Inborn’ if the neonate was delivered inside the study hospitals, ‘out born’ if the neonate was delivered in either health facility or home outside the study hospital. We categorized into ‘Yes’ if inborn or ‘No’ if out born.

Place of death was defined as the place where the neonate died after included in the study, which was categorized into ‘hospital’ if the neonate died inside the hospital where he/she delivered, ‘Home’ if he/she died after discharged from the given hospital, ‘other health facility’ if the neonate died in other health facility other than the given hospital.

Birth type was initially categorized into ‘single’, ‘twin’, ‘triple and above’. Yet for the purpose of analysis twin and triple birth were merged into ‘Multiple births’. Mothers who gave twin and triple birth were counted separately for each birth for the sake of analysis.

Distance was recorded in continuous scale, but later categorized in to <5 km far from the nearest health facility, 5–10 km and > =10 km far from the nearest health facility.

Mode of delivery is classified into ‘spontaneous vaginal delivery’, ‘operative (instrumental) vaginal delivery’ and ‘cesarean section’. Instrumental delivery was defined as the application of either forceps or a vacuum device to assist the mother in effecting vaginal delivery of a fetus.

### Immediate (Intervention) factors

The variables considered in this category were: Hypothermia in which it was classified according to WHO recommendations into ‘Yes’ if body temperature below the normal range (36.5 °C–37.5 °C), ‘No’ if it is within the normal range and above [30].

Temperature control for the hypothermic neonates categorized into: ‘Yes’ if the hypothermic neonate temperature returns to normal after appropriate interventions was undertaken and ‘No’ if the hypothermic neonate temperature is not returned to normal range after the intervention. We did not consider this variable in the Cox-regression model due to large missing values.

Exclusive breast feeding (EBF) (Yes/No): was defined as feeding with only breast milk and nothing else, with the exception of vitamin supplements and prescribed medicines.

Maternal complication categorized into ‘yes’ or ‘No’, which is considered present if the mother had an obstetric hemorrhage, puerperal sepsis and pyrexia, prolonged labor, eclampsia and preeclampsia, mal-presentation and mal-position, premature rupture of membrane (PROM), cord prolapse, obstructed labor, cephalopelvic disproportion (CPD), emergency cesarean section, and retained placenta.

Neonatal complication is considered presented if the neonate had asphyxia, prematurity, infection, hypothermia, jaundice and other rare complications during birth.

### Outcome variable

Early neonatal mortality rate (ENM) was defined as the probability of dying before 7 completed days of life. Late neonatal mortality rate (LNM) as the probability of dying between 7 completed days and before 28 completed days; and overall NM was defined as death of a newborn within 28 days of his/her birth. NMR was defined as the number of deaths of neonates per 1000 live births. NM was categorized into ‘Yes’ if the neonate died ‘No’ if the neonate censored.

Censored births were the neonates who were alive at the end of follow up, withdrawal and lost-to-follow-up. Time-to-death was defined as death of a neonate at specific time (day) within the 28 days follow-up time.

The outcome of the follow up was categorized into ‘death’ if he/she died during follow up, ‘lost to follow up’ if the mother phone was not working, moves to another area from the original place of residence and not met by health extension worker. ‘Withdrawal’ if the mother refuses the follow up due to inconvenience and ‘alive’ if the neonate survival is assured by the data collector at the last follow- up time.

### Data management and analysis

Death of neonate was the event of interest and the coding were “1” for death and “0” for censored. Time-to-death was calculated by subtracting the date of death from birth.

Data was entered, cleaned, recoded and analyzed using STATA version-11.1. For description, frequencies, percentages, and rates, Kaplan Meier curves were used to estimate survival time. Log-rank test was used to look statistical differences among/between the categories of variables. To identify potential predictors of NM, Cox-proportional hazard regression model was fitted by successive stepwise backward elimination. Statistical significance was declared at p-value <0.05. Confounding and effect modification was checked by looking at regression coefficient change if greater than or equal to 15 % and multi-collinearity was checked using variance inflation factor and value of <10 was used as a cutoff point, indicating no colinearity.

Proportional hazard assumption was tested by using covariate specific Proportional hazard assumption test. Residuals were checked using goodness-of-fit test by Cox Snell residuals.

### Ethical clearance

Ethical clearance was obtained from Mekelle University, College of Health Sciences Ethical review board. Permission letters were also sought from Tigray Regional Health Bureau. Written Informed consent from the mother was obtained after clear explanation of the purpose of the study. Confidentiality and anonymity was maintained.

## Results

### Response rate and sociodemographic characteristics

**Fig. 1:**
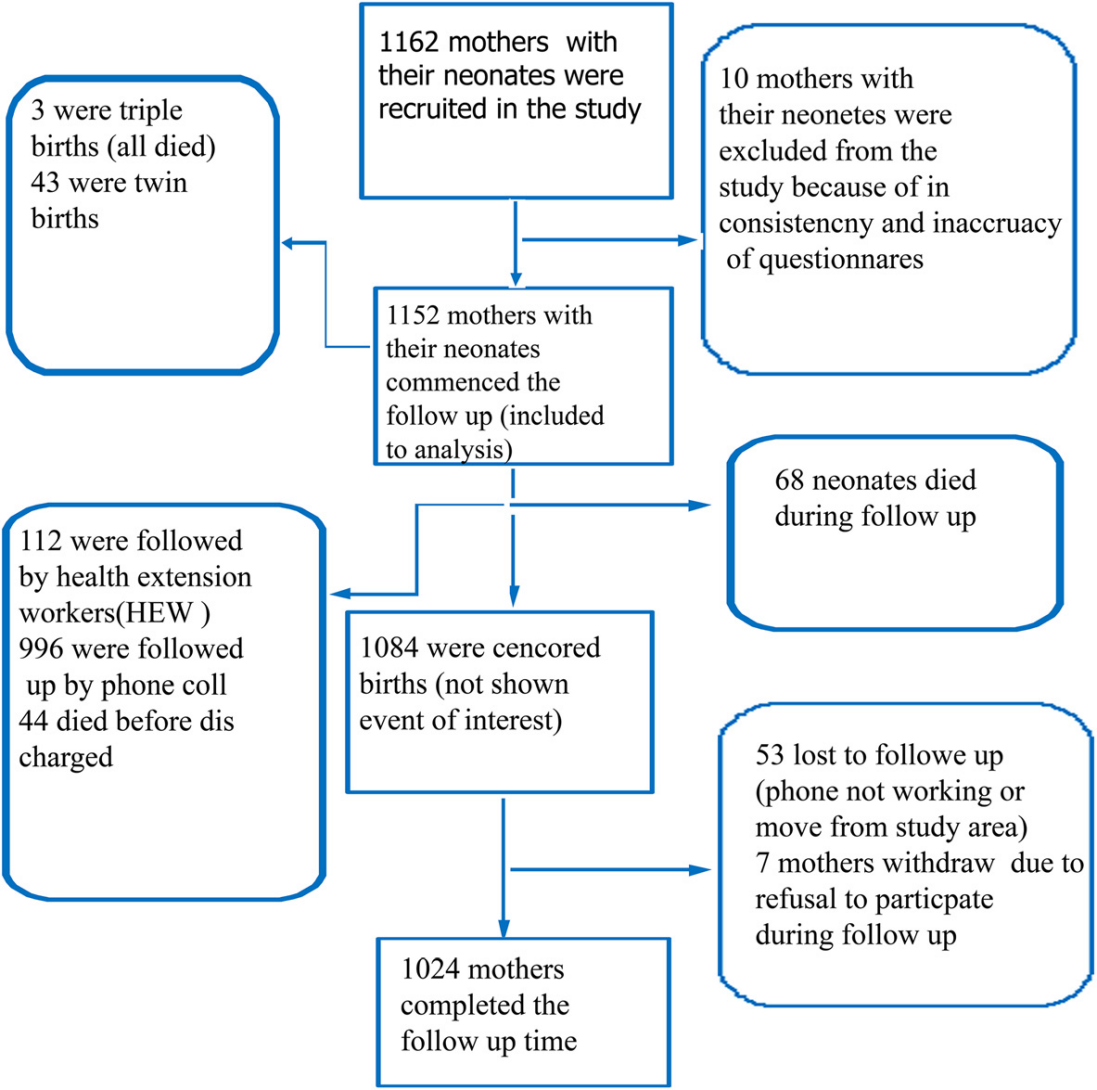
Flow diagram of the overall recruitment and follow up process of mothers and neonates in Tigray region, northern Ethiopia, April–July, 2014. In this figure *Health extension worker* (HEW) means a female completed 10^th^ grade education and received one year training in providing prevention services including breastfeeding, safe and clean delivery, basic antenatal and post-neonatal care. The topics include immunization, family planning, and management of childhood illnesses. The HEWs serves communities in which they reside

Response was obtained from all participants (*n* = 1162), however, ten (0.96 %) observations were excluded from the analysis due to incompleteness (Fig. 1). Of these (*n* = 1152) mothers, 1071 (92.97 %) were married, 368 (31.94 %) of the live births were from teenage mothers (below the age of 18 years), nearly one third 361 (31.34 %) of the mothers had completed secondary school and 373 (36.73 %) of the mothers had a reported monthly income >1500 ETB (Table 1).

**Table 1:** Socioeconomic and demographic characteristics of mothers who gave live births in the randomly selected hospitals of Tigray region, northern Ethiopia, April-July, 2014 (*n* = 1152)

Characteristics	No.	Percent
Hospital		
Ayder Referral Hospital	139	12.1
Adwa Hospital	198	17.2
Lemlem Karl Hospital	147	12.8
Suhl Hospital	143	12.4
Kahsay Abera Hospital	102	8.8
Kidist maram Hospital	213	18.5
Adigrat Hospital	210	18.2
Age at marriage		
< 18 year	68	31.9
≥ 18 year	784	68.1
Religion		
Orthodox	1084	94.1
Other	68	5.9
Residence		
Rural	397	34.5
Urban	755	65.5
Educational status		
Unable to read	275	23.9
Primary	338	29.3
Secondary	361	31.3
Tertiary	178	15.5
Monthly income		
Poor	327	32.19
Medium	316	31.1
Rich	373	36.71
Marital status		
Married	1071	93.0
Other	81	7.0

Rich: ≥1500 Ethiopian birr (ETB), Medium: 600–1500 ETB, Poor: <600 ETB

### Maternal and pregnancy characteristics

Among the total mothers recruited into the study, 172 (14.9 %) of the mothers had previous history of abortion and 91 (7.9 %) of the mothers had a history of stillbirth. The mean and standard deviation of the current age of mothers was 26.65 (sd = ± 5.4).

Regarding medically diagnosed diseases, 100 (8.7 %) had medically known disease. Of these, hypertension had highest prevalence 26 (26 %). But some mothers had two medically diagnosed diseases at the same time, for instance, both hypertension and HIV, for this reason the ‘disease others’ category instead of comprising 68 mothers, it became 73 mothers. It was due to 5 mothers that had double burden of disease simultaneously (Table 2*)*.

**Table 2:** Characteristics of mothers who gave live births in the randomly selected hospitals of Tigray region, northern Ethiopia, April -July, 2014 (*n* = 1152)

Characteristics	No.	Percent	Characteristics	No.	Percent
Age at first birth			Parity		
< 20 year	382	33.3			
20–24 year	551	47.9	None	561	48.7
> 24 year	216	18.8	One	147	12.8
Current age of mother			Two	175	15.2
< 20 year	95	8.2	≥ Three	269	23.3
20–24 year	328	28.5	History of abortion		
25–34 year	590	57.2	Yes	172	14.9
≥ 35 year	139	12.1	No	980	85.1
Number of children			Reason of abortion (*n* = 172)		
Primiparae	610	53.0	Spontaneous	138	80.2
2–4 children	424	36.8	Self initiated	13	7.6
Multiple (≥5)	118	10.2	Induced	21	12.2
Birth type			Number of abortion		
Single	1057	91.7	One	147	85.5
Multiple^b^	95	8.3	Two	19	11.0
Gravidity			≥ Three	3.49	3.5
No pregnancy	475	41.2	History of still birth		
One pregnancy	191	16.6	Yes	91	7.9
Two pregnancy	170	14.8	No	1061	92.1
≥ Three pregnancy	316	27.4	Number of still birth		
Birth interval			One	69	75.8
Not applicable	537	46.6	Two	19	20.9
< Two years	127	11.0	> Two	3	3.3
> Two years	488	42.4	Diagnosed disease		
Ever use of FR^a^			Yes	100	8.7
Yes	650	56.42	No	1052	91.3
No	502	43.58	Type of disease (*n* = 100)		
Method used (*n* = 650)			Hypertension	26	26
Oral	76	11.7	Hemorrhage	6	6
Implant	97	14.9	Other	68	68
Injection	461	70.8			
Other	17	2.6			
Rhesus immunization status			Disease others (*n* = 73)		
Positive	989	85.8	HIV	31	42.5
Negative	163	14.2	TB	7	9.6
Maternal complications			Malaria	6	8.2
			Other	29	39.7
Yes	221	19.2	Tetanus injection		
No	930	80.8	Yes	998	86.6
Type of complication^c^ (*n* = 220)			No	154	13.4
APH	22	10	Number of Tetanus injection		
PPH	30	13.6	One injection	93	9.3
PIH	24	10.9	≥ Two injection	908	90.7
Prolonged/Obstruct	36	16.4	Mortality of older sibling		
CPD	20	9.1	Yes	82	7.1
PROM	12	5.4	No	1070	92.9
Mal presentations	18	8.2	Assistance during delivery		
Other	58	26.4	Midwife	830	72.1
Antenatal utilization			Gyn/obstetrician	148	12.8
Yes	1131	98.2	Emergency Surgery	138	12.0
No	21	1.8	Other	36	3.1
Who attend			Mode of delivery		
Skilled worker	1096	96.9	Vaginal	846	73.4
Other	35	3.1	Cesarean section	241	20.9
No of antenatal utilization			Instrumental	65	5.7
≥ 4 Visits	823	74.1			
< 4 Visits	287	25.9			

^a^Family planning

^b^All multiple births were counted separately

^c^ *APH*, Ante Partum Hemorrhage; *PPH*. Post partum Hemorrhage; *PIH*, Pregnancy induced hypertension; *Gyn*, Gynecologist; *Obstruct*, Obstructed labor; *CPD*, Cephalo Pelvic Disproportion; *PROM*, Premature Rupture of Membrane; *HIV*, Human Immuno Deficiency virus; *TB*, Tuberculosis

### Neonatal and health service characteristics

Regarding the neonatal characteristics, the female to male ratio was 1:1.1. The mean and standard deviation of birth weight and gestational age were 3058 (sd = ±588) and 273 (sd = ±14. 3) respectively. Of the total, 1012 (87.8 %) neonates received EBF and 180 (15.6 %) were large for gestational age neonates. In our study, only few 67 (5.8 %) neonates were outside the study hospitals (out bearing). Among all respondents, 836 (76.3 %) of the mothers were living in <5 km distance to the nearest health facility (Table 3)*.*

**Table 3:** Neonatal and health service related characteristics of neonates delivered in randomly selected hospitals of Tigray region, Northern Ethiopia, April-July, 2014 (*n* = 1152)

Characteristics	No	Percent	Characteristics	No	Percent
Sex			Apgar 5-min score		
Male	610	52.9	Severe (≤3)	8	0.7
Female	542	47.1	Moderate (3–6)	45	4
Exclusive breast feed			Normal (≥7)	1081	95.3
Yes	1012	87.8	Birth defect		
No	140	12.2	Yes	14	1.2
Resuscitation			No	1138	98.8
Yes	154	13.4	Neonatal length (*n* = 1126)		
No	998	86.6	< 46 cm	151	13.4
Apgar 1-min score			46–56 cm	914	81.2
Severe (≤3)	19	1.7	> 56 cm	61	5.4
Moderate (3–6)	166	14.6	Birth weight		
Normal (≥7)	950	83.7	< 2500 gm	121	10.5
Neonatal complications			2500–3500	853	74.3
Yes	126	10.9	> 3500 gm	175	15.2
No	1026	89.1	Gestational age		
Temperature control (*n* = 61)			Preterm	93	8.1
Yes	59	97	Term	1054	91.9
No	2	3	Weight for Gestational Age		
Hypothermic			Appropriate	846	73.5
Yes	61	5.3	SGA	125	10.9
No	1091	94.7	LGA	180	15.6
Neonate inborn or out born			Distance		
Yes	1085	94.2	≤ 5 km	836	76.3
No	67	5.8	5–10 km	175	16.0
			> 10 km	84	7.7

*SGA* small for gestational age, *LGA* large for gestational age, *LBW* low birth weight

### Survival analysis and outcome of the follow up

**Fig. 2:**
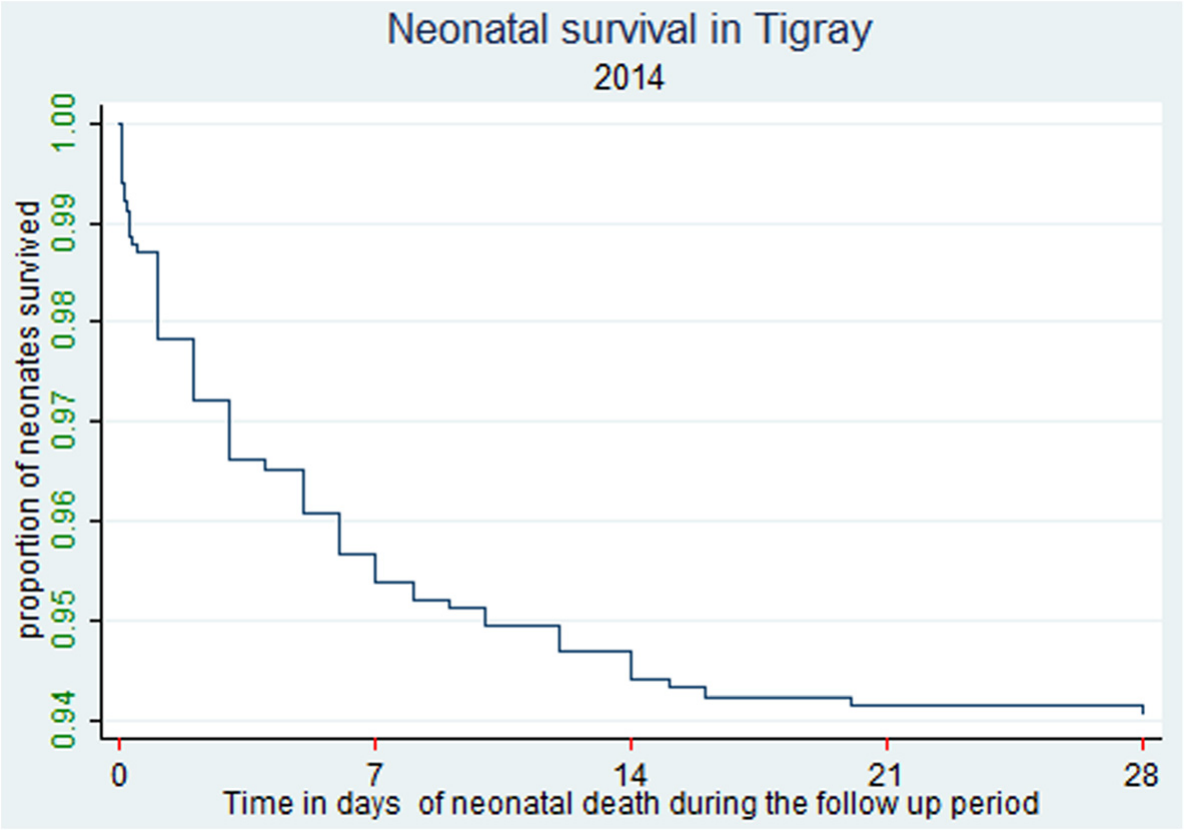
Summary of Kaplan Meir survival estimate on the survival time of neonates born in Tigray region, northern Ethiopia, April–July, 2014 (*n* = 1152). The graph shows the proportion of neonates who survived during the follow up time (birth, 7, 14, 21 and 28 days). Accordingly, as can be seen from the graph; during the first seven days the graph went down gradually which shows a higher proportion of neonates were dying and there was a lower probability of survival. While, over the next 7 days (7 and 14), the proportion of neonates survived has slightly increased and the graph fell down slowly up to the third follow up time (21 day). In the last follow up period the graph became straight which indicates the proportion of neonates survived remained stable indicating virtually no deaths

Neonates were followed up for 27357.508 neonate-days. In the study, 22.05 % of neonatal deaths occurred in the first 24 hours, 47.0 % in the next 3 days and 73.5 % of the neonatal deaths occurred within 7 days. The remaining 26.5 % died in the next 14 days. The cumulative survival rate of neonates at the end of the follow up were 93.96 % (95 % CI: 92.38, 95.21) (Table 4 and Fig. 2)*.* Overall, in this study 68 (6.2 %) neonates died which makes the NMR 62.5 per 1000 live births. At the end of follow up, 1024 (88.9 %) were alive, 53 (4.6 %) lost to follow up and 7 (0.6 %) were withdrawal (Fig. 1). The leading cause of death were 23 (34 %) premature and low birth weight and 21 (31 %) asphyxia, 8 (12 %) infections, 5 (7 %) congenital abnormality and 11 (16 %) died due to other causes. One third (33 %) of the neonates died at home after discharged from the respective hospitals, while 44 (64.7 %) died in the study hospitals and 2 (2.9 %) died in other health facility.

**Table 4:** Survival analysis of neonates during follow up time in the randomly selected hospitals of Tigray region, northern Ethiopia, April-July, 2014 (*n* = 1152)

Interval	Total	Deaths	Lost	Survival probability %	95 % CI
0–1 day	1152	15	1	98.70	97.8, 99.2
1–7 day	1136	35	3	95.65	94.3, 96.7
7–14	1098	11	31	94.68	93.2, 95.8
14–12 day	1056	6	12	94.14	92.6, 95.4
21–28 day	1038	0	9	94.14	92.6, 95.4
28 day	1029	1	1028	93.96	92.4, 95.2

*CI* confidence interval

### Bivariate and multivariate analysis

The log rank test results showed that, survival pattern or time to neonatal mortality has significantly varied over the categories of: birth weight (*X*^2^ for Log rank test =135.9, *p* = 0.00), place of residence (*X*^2^ = 13.1, *P* = 0.0003), number of children (*X*^2^ = 12.5, *P* = 0.0019), history of stillbirth (*X*^2^ = 17.1, *P* = 0.00), history of abortion (*x*^2^ = 9.7, *P* = 0.0019), birth type (*X*^2^ = 29.1, *P* = 0.00), maternal complication (*X*^2^ = 42.3, *P* = 0.00), EBF (*X*^2^ = 275.6, *P* = 0.00), neonatal complication (*X*^2^ = 316.1, *P* = 0.00), weight for GA (*X*^2^ = 19.5, *P* = 0.00), hypothermic birth, 1 and 5 min Apgar score, length of neonate, birth defect, distance to nearest health facility, delivery assistance and place of delivery (Table 5)*.*

**Table 5:** Log rank test result of variables for neonates born in randomly selected hospitals of Tigray region, northern Ethiopia, April –July, 2014 (*n* = 1152)

Variable	Log rank (*X*2)	*P*-value	Variable	Log rank (*X*2)	*P*-value
Hospital	6.05	0.418	Tetanus injection	2.17	0.1407
Age at current birth	3.53	0.3163	Rh immunization	0.28	0.5983
Religion	0.28	0.5943	Mortality older sibling	17.26	0.000
Residence	13.09	0.0003	Sex	0.53	0.4661
Educational status	7.1	0.0687	Exclusive breast feed	275.62	0.000
Occupation	5.8	0.2149	Time of breast feed	2.7	0.1004
Income	2.87	0.2387	Place of death	41.2	0.000
Marital status	0.76	0.383	Antenatal utilization	3.06	0.0802
Number of children	12.51	0.0019	Distance	32.11	0.000
Birth type	29.11	0.000	Mode of delivery	5.44	0.0658
Gravidity	6.8	0.0787	Place of birth	67.75	0.000
Birth interval	3.79	0.1502	Delivery Assistant	9.38	0.0246
Family planning	3.49	0.0619	Birth weight	135.93	0.000
Parity	6.38	0.0947	Gestational Age	98.68	0.000
History of abortion	9.66	0.0019	Length of the neonate	33.67	0.000
History of still birth	17.11	0.000	1-min Apgar score	475.4	0.000
Diagnosed disease	0.73	0.3922	5-min Apgar score	890.8	0.000
Maternal complication	42.29	0.000	Birth defect	30.38	0.000
Temperature control	165.67	0.000	Neonatal complication	316.14	0.000
Weight for GA	19.49	0.000	Hypothermia	176.58	0.000

Variables with P-value <0.05 were prospective candidates for the final model

Multivariable analysis revealed that a neonate born with normal birth weight (2500–3500 gm) had 55 % lesser hazard of neonatal death compared to neonates born with a birth weight of less than 2500 gm (low birth weight) (AHR:0.45, 95 % CI: 0.24, 0.84). Neonates who were not initiated EBF had 7.5 times higher hazard of death than neonates who were initiated EBF (AHR: 7.5, 95 % CI: 3.77, 15.60). Neonates born from mothers who did not have complications were 63 % less likely to die than neonates born from mother’s who had complications (AHR: 0.37, 95 % CI: 0.22, 0.63). Neonates born from mothers residing in ≥10 km far from their nearest health facility had 2.5 times higher to die than neonates born from mothers residing within ≤5 km distance (AHR: 2.5, 95 % CI: 1.29,4.91). Neonates who had no complication after birth had 99.86 % lesser hazard of death than who had the complication (AHR: 0.14, 95 % CI: 0.07, 0.29) (Table 6)*.*

**Table 6:** Multivariate Cox-proportional hazard model of predictors of neonatal mortality, delivered in randomly selected hospitals of Tigray region, northern Ethiopia, April-July, 2014 (*n* = 1152)

Characteristics	Unadjusted HR	95 % CI	Adjusted HR	95 % CI
Residence				
Urban	0.42	0.26,0.68		
Rural (ref)	1.00			
Number of children				
Prime (ref)	1.00			
2–4 children	0.73	0.41,1.29		
Multiple > =5	2.3	1.26,4.23		
Birth type				
Single (ref)	1.00			
Multiple	4.97	2.32,6.97		
History of abortion				
Yes	0.44	0.26,0.75		
No (ref)	1.00			
History of still birth				
Yes	0.31	0.17,0.55		
No (ref)	1.00			
Delivery complication				
Yes (ref)	1.00		1.00	
No	0.23	0.17,0.37	0.37	0.22,0.63
Exclusive breast feed				
Yes (ref)	1.00		1.00	
No	21.63	12.82,36.49	7.5	3.77,15.6
Distance to health facility				
≤5 km (ref)	1.00		1.00	
5–10 km	3.14	1.76,5.58	1.25	0.66,2.37
≥10 km	4.64	2.42,8.89	2.5	1.29,4.91
Mode of delivery				
Spontaneous Vaginal (ref)	1.00			
Cesarean section	1.17	0.654,2.1		
Instrumental	2.38	1.12,5.05		
1-min apgar score				
Severe asphyxia (≤3) (ref)	1.00			
Moderate asphyxia (3–6)	0.13	0.071,0.246		
Normal (≥7)	0.01	0.0049,0.02		
5-min apgar score				
Severe asphyxia (≤3) (ref)	1.00			
Moderate asphyxia (3–6)	0.2	0.088,0.45		
Normal (≥7)	0.0055	0.0023,0.012		
Birth weight				
<2500 gm	11.49	4.49,29.39	1.00	
2500–3500 gm (ref)	1.00		0.45	0.24, 0.84
>3500 gm	1.19	0.46,3.08	0.73	0.26,2.07
Neonatal complication				
Yes (ref)	1.00		1.00	
No	0.04	0.024,0.068	0.14	0.07,0.29
Weight for GA adequacy				
Adequate (ref)	1.00			
SGA	3.16	1.81,5.50		
LGA	1.02	0.49,2.11		
Hypothermia				
Yes (ref)	1.00			
No	0.075	0.046,0.12		

*HR* hazard ratio, *GA* gestational age, *SGA* small for gestational age, *LGA* large for gestational age

**Fig. 3:**
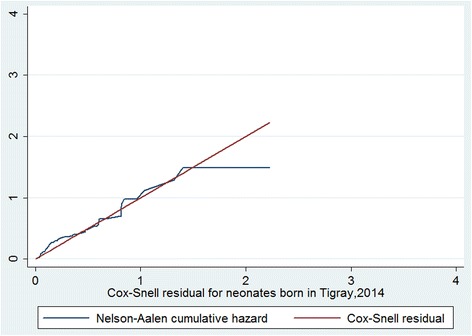
Cox-Snell residual Nelson -Alen cumulative hazard graph on neonates born in Tigray region, Northern Ethiopia, April -July 2014. This figure shows if the Cox regression model fits the data, these residuals should have a standard censored exponential distribution with hazard ratio. The hazard function follows the 45° line very closely

No confounding, effect modification and multi-collinearity were observed in this stud*y.* No covariates violated the proportional-hazard assumption test. For the residual test, it was possible to conclude that the final model fits the data very well (Fig. 3).

## Discussion

In this study, high NMR (62.5 per 1000 live births) was observed, which is striking because the deaths happened in hospitals where highly skilled birth attendants and formal health care system are present. This rate is higher than several studies conducted in Ethiopia and Africa [9, 11, 14, 31–34] and the national NM, EDHS 2011 report [4]. The discrepancy could be explained in terms of study design difference and the place where information was gathered, in which most of the mothers in our study and other hospital based studies [35] probably came very late with serious obstetric complications and may overestimate the community NM. However this could also be due to actual differences in rate of NM. Regardless, our data provided evidence as NM remains high in the study setting and the reasons remain uncertain. It is also much higher than the regional Health Management Information System report, which had reported that 20 neonatal deaths within five months period [21]. This can clearly show underreporting of neonatal mortalities for retrospective surveys and misclassification of stillbirths with ENM [3, 9]. This phenomenon is also similar to studies conducted in urban Pakistan and Africa, which showed a good access to health service and attendance by a skilled health professional were found to have high NM [36, 37], respectively. Hence, in our study settings, services being rendered to mothers and neonates seemed substandard, which is unacceptable in areas where services are attended by skilled birth attendants.

The incidence of ENM was higher than the LNM and the overall incidence is almost two times higher than the study findings of Butajira, Ethiopia [12]. This difference might be attributable to actual high incidence of NM in our study setting. It can also be because of the study area in which the Butajira study was conducted in a rural community, which was followed up for a longer period of time. However, the data for this study was collected from hospitals where mothers may have been diagnosed with complications. Despite the fact that we considered these possibilities, there is an intolerable high incidence of NM in our study setting.

Our study has revealed that most neonatal deaths occurred in the first 24 h and in the first week of life, which is consistent with different studies conducted before [11, 12, 27, 37, 38]. Other studies reported that most neonates died in their first week of life because of complication occurring during pregnancy and birth [1–3]. Poor quality of antenatal care, delay in identification and poor management of complications during pregnancy and birth by health workers might be the possible reasons.

The current study finding revealed that neonates with a low birth weight experienced higher mortality. This finding is similar to a study conducted in different parts of developing countries [3, 14, 17, 34, 38, 39]. Low birth weight contributes primarily from the mother’s poor health and nutrition [40]. Improving obstetric care is crucial, which can reduce the incidence of low birth weight [41]. In this study, all triplets and most of twin births died, as low birth weight could be the result of preterm delivery or small size for gestational age. This could have happened because of poor feeding practices of mothers, as well as subsequent malnutrition, inadequate warmth and lack of breastfeeding.

Our findings showed that neonates born from mothers who have complications during birth had a higher chance of death. This is similar to previous studies reported on maternal complications related to high risks of NM [3, 16, 27, 34, 42]. However, recent studies from Africa [37] and Indonesia [43] showed that having delivery complications during birth have a protective effect on NM. Hence, these studies indicated that managing complications are possible, which may be related to the focus of skilled health professionals on mothers that have complications. This study considered the common maternal complications as the reasons for NM. This may be due to the quality and availability of services, including emergency obstetric services [7]. Weak integrated efforts in recognition of mothers with complications and delayed referrals by health workers could also be other reasons. This is supported by some studies’ findings, which indicated that high NM often occurs because of delivery and pregnancy-related complications and denotes the quality of healthcare in an area [10].

In accordance with our findings, in general, studies show that male-presentation, prolonged labor, hemorrhage, infection, PROM and obstructed labor were associated with NM [3, 14, 27, 36, 39]. Accordingly, saving the mother and managing these complications could improve survival of neonates in our study setting.

Distance to the nearest health facility was also among the independent predictors of NM. Similarly, several studies also reported that neonates who live in remote and hard to reach areas from health facilities are at increased rate of death than those who live in nearby to the health facilities [12, 17, 28, 37, 39]. This can be explained in terms of lack of access to health service during pregnancy, which resulted in lower attendance rate at antenatal and delivery because of the distance [17]. It can be associated with missing opportunities to prevent and treat intra-partum complications and these circumstances may cause intra-partum complications and subsequent ENM.

The current study findings revealed that initiating EBF has a protective effect on hazards of NM. This is also reported by some studies, as EBF is essential to reduce newborn deaths [44]. Other studies reported that risk of NM delayed initiation of breastfeeding, where the risks were four times higher in children who were given other fluids in addition to breastmilk [45]. A study in sub-Saharan African countries demonstrated that NM could be reduced by 16 % if mothers started breastfeeding from Day 1 and 22 % if they started within the first hour [46]. Similarly, in our study, all neonates who did not initiate EBF had not initiated breastfeeding within the first one hour. Early initiation of breastfeeding is necessary to save newborn lives [24]. However, in our study, initiation of EBF seemed to be inadequately practiced and promoted. But it may be necessary to be cautious in interpreting this finding as sometimes neonates that are the most sick might be the least likely to be able to breastfeed compared to healthier neonates.

Neonatal complications are also one of the independent predictors for NM. In this study, the main causes of neonatal complications were: meconium aspiration, prematurity, low birth weight and prolonged labor, which lead to Apgar score less than 7 and birth asphyxia. The causes of complications result in neonatal asphyxia, hypothermia, sepsis and other complications, although there are no significant differences among them. Similarly, reports indicate that complications of prematurity are currently among the leading causes of NM [1, 2, 14]. A study conducted in South Africa revealed that neonatal complications were the causes for NM and morbidity like asphyxia and hypothermia [47]. This might be because of delay in identification of newborn complications, poor management and lack of postnatal care in our study settings.

The strength of this study is that it had included all predictors of NM and data collection was also complete and reliable. The study area covers the entire region of Tigray; it is generalizable to all hospitals of the region and Ethiopia. Limitations of this study are that the effect of seasonal variation on NM was not considered and the study was conducted only in public health institutions. Moreover, home deliveries and neonates who arrived at study hospitals after 6 h of birth were not considered as part of the study, as we failed to track the deaths that occurred at home and this may underestimate the NM rate because home deliveries are at risk of complications and deaths. We encountered 5.2 % lost to follow up and withdrawals because mothers were unable to answer phone calls, meet with health extension workers and/or are not available during physical visits. Most of the mothers were moved from the original place of residence and their phone was not working during the follow up time. These mothers may encounter neonatal deaths that could underestimate our neonatal mortality rate.

## Conclusion

In this study, unacceptably high burden and incidences of NM (lower survival) was observed, which is unexpected as the births were attended by highly skilled health workers. Moreover, proximate factors (maternal, neonatal and health service related factors) were the important predictors.

Therefore, a comprehensive effort should be undertaken to improve health service provided in the region, especially on the competency of health workers and quality of health services, including availability of necessary equipment and setup to manage neonatal and maternal complications. In addition, maternal health before delivery, including quality of antenatal care utilization, low birth weight, initiating exclusive breast feeding, continuum care of the neonate after discharge and access should be considered to improve survival time of neonates.

## Abbreviations

AIDS, acquired immuno deficiency syndrome; BMI, body mass index; CPD, cephalo pelvic disproportion; EBF, exclusive breast feeding; ENM, early neonatal mortality; ETB, Ethiopian birr; HIV, human immunodeficiency virus; LNM, late neonatal mortality; NM, neonatal mortality; NMR, neonatal mortality rate; PROM, premature rupture of membrane; U5M, under-five-mortality; WHO, World Health Organization

## References

[1] UNICEF, World Health Organization, the World Bank, the United Nations Population Division: Levels & Trends in Child Mortality: Estimates Developed by the UN Inter-agency Group for Child Mortality Estimation (UN IGME). UN IGME; 2014.

[2] UNICEF, World Health Organization, the World Bank, the United Nations Population Division: Levels & Trends in Child Mortality: Estimates Developed by the UN Inter-agency Group for Child Mortality Estimation (IGME). UN IGME; 2013.

[3] Lawn JE, Cousens S, Zupan J. Lancet neonatal survival steering team. 4 million neonatal deaths: when? where? why? Lancet. 2005;365(9462):891–900. 10.1016/S0140-6736(05)71048-5 15752534

[4] Central Statistical Agency [Ethiopia] and ICF International (2012). Ethiopia demographic and health survey 2011.

[5] News from the last 10 kilometers. Ethiopia achieves millennium development goal on reducing child mortality!.Ethiopia: An L10K publication 2013; 2(1). Available from: http://www.10k.jsi.com. Accessed Aug 2014.

[6] UNICEF. In Ethiopia, a far reaching health worker programme has helped reduce child mortality across the country. 2013. Available from: http://www.unicef.org/infobycountry/ethiopia_70372.html.. Accessed Apr 2014.

[7] Kayongo M, Rubardt M, Butera J, Abdullah M, Mboninyibuka D, Madili M (2006). Making EmOC a reality—CARE’s experiences in areas of high maternal mortality in Africa. Int J Gynecol Obstet.

[8] Mekbib T, Kassaye E, Getachew A, Tadesse T, Debebe A (2003). Averting maternal death and disability, the FIGO save the mothers initiative: the Ethiopia–Sweden collaboration. Int J Gynecol Obstet.

[9] Oestergaard MZ, Inoue M, Yoshida S (2011). Neonatal mortality levels for 193 countries in 2009 with trends since 1990: a systematic analysis of progress, projections, and priorities. Plos Med.

[10] Lawn JE, Blencowe H, Pattinson R (2011). Stillbirths: where? when? why? how to make the data count?. The Lancet.

[11] Gill CJ, Phiri-Mazala G, Guerina NG (2011). Effect of training traditional birth attendants on neonatal mortality (Lufwanyama Neonatal Survival Project): randomized controlled study. BMJ.

[12] Muluken G, Mitike M, Wubegzier M (2014). Trends and risk factors for neonatal mortality in Butajira District, South Central Ethiopia, (1987–2008): a prospective cohort study. BMC Pregnancy Childbirth.

[13] Jahan S (2008). Poverty and infant mortality in the Eastern Mediterranean region: a Meta analysis. J Epidemiol Community Health.

[14] Rahman S, Ansari EL, Oliver CE, Karen OP (2012). Neonatal mortality: incidence, correlates and improvement strategies. INTECH.

[15] Rahman S, Salameh K, Bener A, Ansarin WE (2010). Socioeconomic associations of improved maternal, neonatal, and perinatal survival in Qatar. Int J Equity Health.

[16] Emmanuel T, Notion G, Gerald S, Addmore C, Mufuta T, Simukai Z (2011). Determinants of perinatal mortality in Marondera district, Mashonaland East Province of Zimbabwe, 2009 a case control study. Pan Afr Med J..

[17] World Data Bank: World Development Indicators. The World Bank Group. 2013. Available from: http://www.worldbank.org/en/country/ethiopia. Accessed March 2014.

[18] Getachew B, Yifru B (2012). Perinatal mortality and associated risk factors: a case control study. Ethiopian J Health Sci.

[19] Darmstadt GL, Bhutta ZA, Cousens S (2005). Evidence-based, cost-effective interventions: how many newborn babies can we save?. Lancet.

[20] Zupan J (2005). Perinatal mortality in developing countries. N Engl J Med.

[21] Tigray Region Health bureaue. Report on the health status of Tigray region. The government of the national state of Tigray; Mekelle. August 2014. Available from: http://www.trhb.gov.et. Acessed Nov 2014.

[22] Yared M, Biruk T, Daniel ST, Tedbabe D, Abeba B (2013). Neonatal mortality in Ethiopia: trends and determinants. BMC Public Health.

[23] World Health Organization (2003). Standard neonatal verbal autopsy questionnaire revised version.

[24] Federal Ministry of Health, Ethiopia (2011). Integrated management of newborn and child hood illness for health workers.

[25] World Health Organization (2007). International statistical classification of diseases and related health problem, 10th revision (ICD-100).

[26] Mosley WH, Chen LC (1984). An analytical framework for the study of child survival in developing countries. Popul Dev Rev.

[27] World Health Organization (1993). International statistics classification of diseases and related health problems.10th revision.

[28] World Health Organization (1995). Physical status: the use and interpretation of anthropometry: report of a WHO expert committee.

[29] Alexander GR, Himes JH, Kaufman RB, Mor J, Kogan M (1996). A United States national reference for fetal growth. Obstet Gynecol.

[30] World Health Organization (1997). Thermal protection of the newborn: a practical guide.

[31] Debelew GT, Afework MF, Yalew AW. Determinants and Causes of Neonatal Mortality in Jimma Zone, Southwest Ethiopia: A Multilevel Analysis of Prospective Follow up Study. PLoS ONE. 2014;9(9):e107184. 10.1371/journal.pone.0107184 PMC4169420 25232842

[32] Yaya Y, Eide KT, Norheim OF, Lindtjorn B. Maternal and Neonatal Mortality in Southwest Ethiopia: Estimates and SocioEconomic Inequality. PLoS ONE. 2014;9(4):e96294. 10.1371/journal.pone.0096294 PMC4005746 24787694

[33] Diallo AH, Meda N, Oue’draogo WT, Cousens S, Tylleskar T, For the PROMISE-EBF study group (2011). A prospective study on neonatal mortality and its predictors in a rural area in Burkina Faso: can MDG-4 be met by 2015?. J Perinatol.

[34] Adetola AO, Tongo OO, Orimadegun AE, Osinusi K (2011). Neonatal mortality in an urban population in Ibadan, Nigeria. Neonatal mortality in Ibadan, Nigeria. Pediatr Neonatol.

[35] Berhan Y, Abdela A (2004). Emergency obstetric performance with emphasis on operative delivery outcome: does it reflect the quality of care?. Ethiop J Health Dev.

[36] Jehan I, Harris H, Salat S (2009). Neonatal mortality, risk factors and causes: a prospective population-based cohort study in urban Pakistan. Bull World Health Organ.

[37] Singh K, Brodish P, Suchindran C (2014). A regional multilevel analysis: can skilled birth attendants uniformly decrease neonatal mortality?. Matern Child Health J.

[38] Baqui AH, Ahmed S, Arifeen SE (2009). Effect of timing of first postnatal care home visit on neonatal mortality in Bangladesh: an observational cohort study. BMJ.

[39] Nankabirwa V, Tumwine JK, Tylleskär T (2011). Perinatal mortality in Eastern Uganda: a community based prospective cohort study. PLoS One.

[40] Skokić F, Bačaj D, Selimović A, Hasanović E, Muratović S, Halilbašić A. Association of Low Birth Weight Infants and Maternal Socio demographic Status in Tuzla Canton during 1992–1995 War Periods in Bosnia and Herzegovina. Int J Pediatr. 2010:789183. 10.1155/2010/789183 PMC3068307 21490700

[41] Onwuanaku CA, Okolo SN, Ige KO, Okpe SE, Toma BO (2011). The effects of birth weight and gender on neonatal mortality in north central Nigeria. BMC Res Notes.

[42] Kusiako T, Ronsmans C, Van der PL (2000). Perinatal mortality attributable to complications of childbirth in Matlab, Bangladesh. Bull World Health Organ.

[43] Titaley CR, Dibley MJ, Roberts CL (2012). Type of delivery attendant, place of delivery and risk of early neonatal mortality: analyses of the 1994–2007 Indonesia Demographic and Health Surveys. Health Policy Plan.

[44] Begum K, Dewey KG. Impact of early initiation of exclusive breastfeeding on newborn deaths. Alive & Thrive technical staff. 2010;1. Available from: http://www.alive and thrive.org. Accessed Nov 2014.

[45] Edmond KM, Zandoh C, Quigley MA, Amenga-Etego S, Owusu-Agyei S, Kirkwood RB (2006). Delayed breastfeeding initiation increases risk of neonatal mortality. Pediatrics.

[46] Edmond KM, Kirkword BR, Amenga-Etego S, Owusu-Ageyei S, HurtL S (2007). Effect of early infant feeding practices on infection specific neonatal mortality: an investigation of the causal links with links with observational data from rural Ghana. Am J Cli Nutr.

[47] Kalimba EM, Ballot DE. Survival of extremely low-birth-weight infants: Department of Pediatrics and Child Health, University of the Witwatersrand and Charlotte Maxeke Johannesburg Academic Hospital, Johannesburg. S Afr J CH. 2013;7(1):13–16.

